# Supplementary material for: Deletion of wfs1 Impairs Oligodendrocyte Precursor Cells Dorsal Distribution and Myelination Through the wfs1-hmgcs1 Axis in Zebrafish
Source: Biology (Basel). 2026 Aug 16;15(16):1402. doi: 10.3390/biology15161402 (PMC13510151; doi:10.3390/biology15161402)
Supplement: Supplementary file 1 [file biology-15-01402-s001.zip › File S1-Transcriptome sequencing data and differentially expressed genes (DEGs) in wfs1-- zebrafish at 5 dpf/2.KEGG/wfs1KOvsWT/ALL/src/dre00970.html]

dre00970


- dre:394188

- Down regulated genes

394188(-1.02302978359784)

- dre:792787

- Up regulated genes

792787(2.16701248433346)

- Down regulated genes

324940\_1(-11.2752233652613)

- dre:101884223

- Up regulated genes

101884223(1.92091516073639)

Close
